# Effect of Sequential Treatment with Bisphosphonates After Teriparatide in Ovariectomized Rats: A Direct Comparison Between Risedronate and Alendronate

**DOI:** 10.1007/s00223-017-0263-6

**Published:** 2017-03-23

**Authors:** Tetsuo Yano, Mei Yamada, Daisuke Inoue

**Affiliations:** 1 Research Institute, EA Pharma Co., Ltd, 1-1 Suzuki-cho, Kawasaki-ku, Kawasaki-shi, Kanagawa 210-8681 Japan; 2 Research Institute for Bioscience Products & Fine Chemicals, Ajinomoto Co., Inc., 1-1 Suzuki-cho, Kawasaki-ku, Kawasaki-shi, Kanagawa 210-8681 Japan; 3 Third Department of Medicine, Teikyo University School of Medicine, 3426-3 Anesaki, Ichihara-shi, Chiba, 299-0111 Japan

**Keywords:** Risedronate, Bone quality, Bone architecture, Alendronate, Teriparatide

## Abstract

Teriparatide (TPTD), a recombinant human parathyroid hormone N-terminal fragment (1–34), is a widely used bone anabolic drug for osteoporosis. Sequential treatment with antiresorptives such as bisphosphonates after TPTD discontinuation is generally recommended. However, relative effects of bisphosphonates have not been determined. In the present study, we directly compared effects of risedronate (RIS) and alendronate (ALN) on bone mineral density (BMD), bone turnover, structural property and strength in ovariectomized (OVX) rats, when administered after TPTD. Female Sprague Dawley rats were divided into one sham-operated and eight ovariectomized groups. TPTD, RIS, and ALN were given subcutaneously twice per week for 4 or 8 weeks after 4 week treatment with TPTD. TPTD significantly increased BMD (+9.6%) in OVX rats after 4 weeks of treatment. 8 weeks after TPTD withdrawal, vehicle-treated group showed a blunted BMD increase of +8.4% from the baseline. In contrast, 8 weeks of treatment with RIS and ALN significantly increased BMD to 17.4 and 21.8%, respectively. While ALN caused a consistently larger increase in BMD, sequential treatment with RIS resulted in lower Tb.Sp compared to ALN in the fourth lumbar vertebra as well as in greater stiffness in compression test. In conclusion, the present study demonstrated that sequential therapy with ALN and RIS after TPTD both improved bone mass and structure. Our results further suggest that RIS may have a greater effect on improving bone quality and stiffness than ALN despite less prominent effect on BMD. Further studies are necessary to determine clinical relevance of these findings to fracture rate.

## Introduction

Teriparatide (TPTD), an N-terminal fragment of human parathyroid hormone (PTH) (1–34), is an anabolic agent for the treatment of osteoporosis [1–3] that markedly increases bone mineral density (BMD) and decreases fracture risk [4–6]. It has been shown to be even more potent particularly in lumbar BMD gains than standard antiresorptives such as alendronate (ALN) and raloxifene [7–10]. However, duration of TPTD therapy is restricted to 2 years during lifetime due to a concern about development of osteosarcoma [11, 12]. Moreover, discontinuation of TPTD treatment results in substantial loss of the BMD gains by the drug. It is thus recommended to sequentially use antiresorptives following TPTD therapy to maintain bone mass [4, 13, 14].

Several studies have investigated efficacy of concurrent or sequential treatment with TPTD and antiresorptives including bisphosphonates [15–23]. Contrary to an initial expectation that simultaneous treatment with anabolic and antiresorptive agents would result in a greater BMD increase in an additive or synergistic manner, a combination therapy of TPTD and ALN turned out to be less efficacious than TPTD monotherapy [16]. Sequential treatment with TPTD after antiresorptives also resulted in diminished effects. The blunted response to TPTD by concomitant or prior treatment with antiresorptives seemed in part due to the fact that the anabolic effect of TPTD depends on bone remodeling activity [23–26]. Exceptionally, however, zoledronate and denosumab showed additive effect when used in combination with TPTD [9, 27] despite their strong antiresorptive activities. Thus, the mechanism of bone anabolic effects of TPTD and its interactions with antiresorptive agents remain to be elucidated.

Risedronate (RIS) and ALN are nitrogen-containing bisphosphonates widely used as first-line drugs for the treatment of osteoporosis. Although these two bisphosphonates share the fundamental mechanism of inhibiting bone resorption [28], they significantly differ in potency of inhibiting farnesyl pyrophosphate synthase and affinity to hydroxyapatite [29]. Such unique properties may explain differences in clinical efficacy of the two bisphosphonates; despite its less prominent effect on bone turnover and BMD than ALN, RIS appears similarly to or even more effective in preventing hip and non-vertebral fractures during the first year of therapy [30] and in experimental animals as well under certain conditions [31]. As for sequential therapy, 1-year TPTD therapy after prior treatment with RIS or ALN for at least 2 years significantly increased BMD and bone formation markers, and the effect was greater in patients previously treated with RIS than in those pretreated with ALN [22]. It is also of note that a combination therapy with RIS and TPTD caused a greater effect than monotherapy in humans [32, 33]. In rodents, sequential treatment with RIS was able to maintain BMD gains by PTH pretreatment in ovariectomized (OVX) animals [34]. Similar results were obtained with ALN when administered after TPTD in rats [35]. It is currently unknown whether effects of sequential treatment with bisphosphonates after TPTD therapy differ among different bisphosphonates.

Thus, in the present study, effects of sequential treatment with RIS and ALN after TPTD therapy were directly compared in OVX rats.

## Materials and Methods

### Experimental Animals

Seventy two female Sprague–Dawley (SD) rats at 7 weeks of age were used (Beijing Vital River, Beijing, China). The animals were housed two per cage in a room with light/dark cycle of 12/12 h and were acclimated to study condition for 5 weeks prior to the start of the experiment. All animals were pair-fed rodent food (irradiated, Shanghai SLAC Laboratory Animal Co. Ltd., Shanghai, China). Water from PharmaLegacy Laboratories Co. Ltd. (Shanghai, China) in house production was available to animals *ad libitum*. The experimental protocols were approved by Animal Care and Use Committee of EA Pharma Co. Ltd. and PharmaLegacy Laboratories IACUC.

### Materials

Risedronate (cat no. R521500) was purchased from Toronto Research Chemical (Toronto, ON, Canada). Alendronate (cat no. 126855) and teriparatide (cat no. 05235501) were purchased from EMD Biosciences (Darmstadt, Germany). Atropine (cat no. A0132), calcein (cat no. C0875), buprenorphine (cat no. B9275), gentamicin (cat no. G1914), and ketamine (cat no. K2753) were purchased from Sigma (St. Louis, MO, USA). Isoflurane (cat no. 1001936040) was purchased from Baxter (Deerfield, IL, USA).

### Experimental Design

**Fig. 1:**
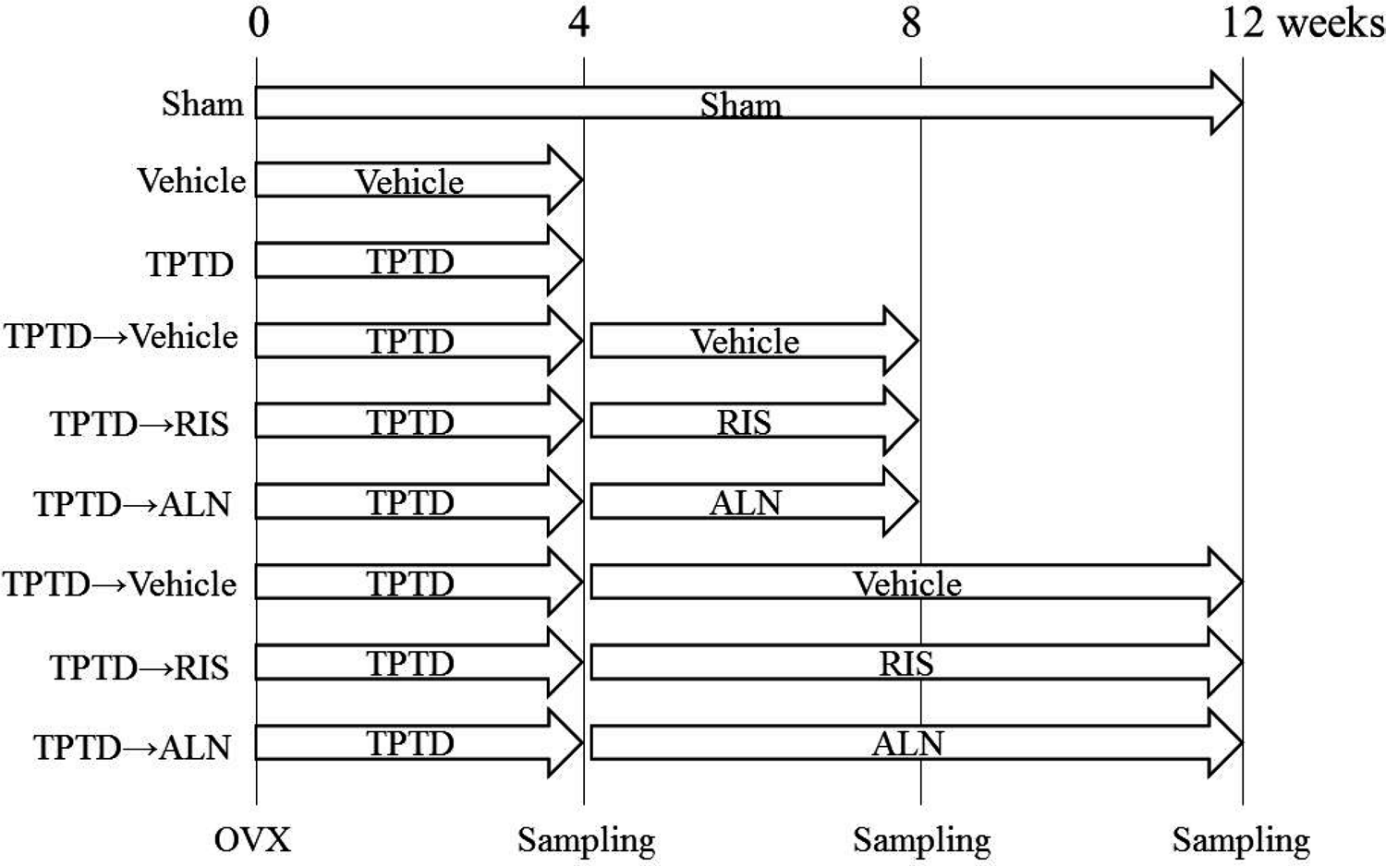
Study timeline. Treatment was started from the next day after surgery. Animals were treated with vehicle or teriparatide (TPTD) twice a week for 4 weeks. After TPTD withdrawal, vehicle, risedronate (RIS), or alendronate (ALN) were given subcutaneously for 4 or 8 weeks. BMD, bone architecture, and bone strength was measured at 4, 8, or 12 weeks after surgery

The experimental timeline is summarized in Fig. 1. The animals were assigned to sham-operated or ovariectomized (OVX) group by matching body weight and bone mineral density (BMD) of lumbar vertebrae (L2-L5) by BioBook system (IDBS, ID Business Solutions LTD., Shanghai, China). The OVX group was assigned to (1) vehicle, (2) TPTD, (3) TPTD and vehicle, (4) TPTD and RIS, (5) TPTD and ALN, (6) TPTD and vehicle, (7) TPTD and RIS, and (8) TPTD and ALN with eight animals in each group. The groups (1) and (2) were sacrificed at 4 weeks; (3), (4), and (5) were at 8 weeks; and (6), (7), (8), and the sham groups were at 12 weeks. TPTD (0.1 mg/kg, s.c.) was injected twice a week for 4 weeks. RIS (0.01 mg/kg, s.c.) or ALN (0.02 mg/kg, s.c.) was injected twice a week for 4 or 8 weeks after TPTD (Fig. 1).

Atropine (0.05 mg/kg, s.c.) was injected before surgery. Under anesthesia with ketamine (70 mg/kg, i.p.) and isoflurane 1–5% inhalation with a 0.8–1.5 L flow rate of oxygen, the animals were removed of the bilateral ovaries or sham-operated from a low abdominal approach. Animals received analgesics including but not limited to buprenorphine (0.05 mg/kg, i.m.) and gentamycin (20 mg/kg, i.m.) after surgery.

Four, 8, and 12 weeks after dosing, the animals were evaluated for BMD of lumbar vertebrae (L2-L5) by in vivo Dual-Energy X-ray Absorptiometry (DEXA, Hologic Discovery SL; Hologic, Inc., Waltham, MA, USA). After sacrifice at the end of each treatment protocol, BMD of the fourth lumbar vertebra was measured ex vivo by peripheral Dual-Energy X-ray Absorptiometry (pDEXA, Norland/Stratec, Pforzheim, Germany). They were also subjected to mechanical testing (L5) and histomorphometric analysis (L4).

### Labeling and Histomorphometry

Fluorochrome dye of calcein was administered to quantify bone formation rates. Calcein (10 mg/kg, s.c.) was administered at 9 and 2 days prior to sacrifice. The L4 were collected and prepared by undecalcifying methods. The specimens were dehydrated through gradient ethanol and embedded in methyl methacrylate. The blocks were sectioned at about 5 μm in thickness for staining and histomorphometry. Static and dynamic histomorphometric analyses of the L4 were performed. One section was stained with Goldner’s trichrome for static parameter measurements, and another was kept unstained for dynamic parameter measurements. The histomorphometry was done using OsteoMeasure™ (OsteoMetrics, Inc., Decatur, GA) software program on a computer in conjunction with a Nikon E501 bright field and fluorescent microscope (Nikon Corporation, Tokyo, Japan) to measure the primary parameters: bone surface (BS, µm), osteoclast surface (Oc.S, μm), osteoclast number (N.Oc), osteoid surface (OS, μm), osteoblast surface (Ob.S, μm), and mineralizing surface (MS, μm). The following parameters were calculated from primary parameters: osteoid surface (OS/BS, %), number of osteoclasts per trabecular surface (N.Oc/BS, /mm), surface area of osteoclasts contacting the trabecular surface (Oc.S/BS, %), osteoblast surface (Ob.S/BS, %), mineralized surface (MS/BS, %), bone formation rate (BFR/BS, %/year), and mineral apposition rate (MAR, μm/day). Osteoclasts were identified as cells forming resorption lacunae at the bone surface. Structural indices were measured by these systems: bone volume (BV/TV, %), trabecular thickness (Tb.Th, μm), trabecular number (Tb.N, /mm), and trabecular separation (Tb.Sp, μm) [36].

### Mechanical Testing

The fifth lumbar vertebrae (L5) and femurs were tested for bone strength by compression and three-point bending tests, respectively, using previously described methods with a slight modification [37]. The biomechanical testing was done using MTS858 (MTS System, Minneapolis, MN). For compression tests, the vertebra was fixed on a holder with adhesive agent and placed on the testing platform. For three-point bending of femur, the span of the two support points below the bone was 14 mm. The deformation rate was 6 mm/min for both femurs and vertebrae.

Load-deformation curves were transported to a personal computer and acquired by Team 490 software (version 4.10, Nicolet Instrument Technologies, Madison, WI). Sigma Plot 7.0 software (SPSS, Chicago, IL) was used to smooth the load-deformation curve and calculate the extrinsic material properties of the bone samples, including the maximal load (N) and linear stiffness (N/mm).

### Statistical Analysis

All data were expressed as mean ± standard deviation (SD). According to the results of Shapiro–Wilk test for normal distribution, group differences were analyzed by *t*-test or Wilcoxon test and analysis of variance (ANOVA) with post hoc Dunnett’s test or Steel test on JMP (ver. 12.0.1, SAS Institute, Tokyo, Japan), and two-way ANOVA on Prism (ver. 6.07, Graph Pad Software, .CA, USA). *P* values less than 0.05 were considered statistically significant.

## Results

### In Vivo BMD of L2-L5 by DEXA

**Fig. 2:**
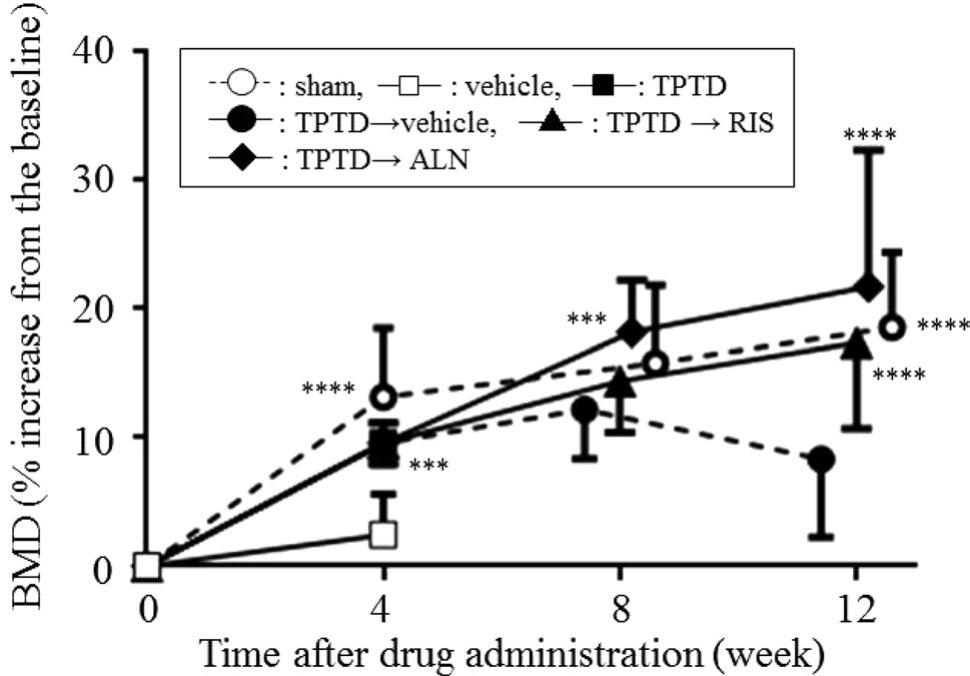
Effect of sequential treatment with bisphosphonates after teriparatide on lumber vertebral BMD (L2-L5). Sham-operated rats are shown in *open circles*. Ovariectomized rats treated with vehicle for 4 weeks are shown in *open squares*. The other rats were first treated with teriparatide for 4 weeks, and then with vehicle (*closed circles*), risedronate (*closed triangles*), or alendronate (*closed rhombuses*) for up to 8 weeks. Percent increases from the baseline in lumber vertebral BMD (L2-L5), measured by in vivo DEXA are shown in mean ± SD. At 4 weeks, sham and vehicle: *n* = 8 and TPTD: *n* = 56. At 8 weeks, sham: *n* = 8 and TPTD→vehicle, TPTD→RIS, and TPTD→ALN: *n* = 16. At 12 weeks, sham, TPTD→vehicle, TPTD→RIS, and TPTD→ALN: *n* = 8. ^***^ *p* < 0.001 and ^****^ *p* < 0.0001 compared with the vehicle-treated group at the same time point

As shown in Fig. 2, the sham-operated rats gradually gained BMD throughout the study: 13.2, 15.8, and 18.6% at 4, 8, and 12 weeks, respectively. The vehicle-treated OVX rats only showed a 2.4% increase in BMD during the initial 4 weeks. In contrast, the TPTD-treated rats showed a significantly greater increase in BMD at 4 weeks (9.6%, *p* < 0.0002, compared with vehicle). After discontinuation of TPTD, the subsequent treatment with vehicle caused a gradual decline in BMD during 4–12 weeks. In sharp contrast, the rats sequentially treated with ALN or RIS following TPTD therapy attained the BMD level similar to the sham-operated animals and significantly higher than the TPTD vehicle-treated OVX rats at 12 weeks. At 8 weeks, only the TPTD-ALN group, showed a significantly higher BMD than that of TPTD-vehicle rats, suggesting that effect of ALN on BMD might be somewhat superior to that of RIS at the early phase of sequential treatment after TPTD.

### Ex Vivo BMD of L4 and Tibia by pDEXA

**Fig. 3:**
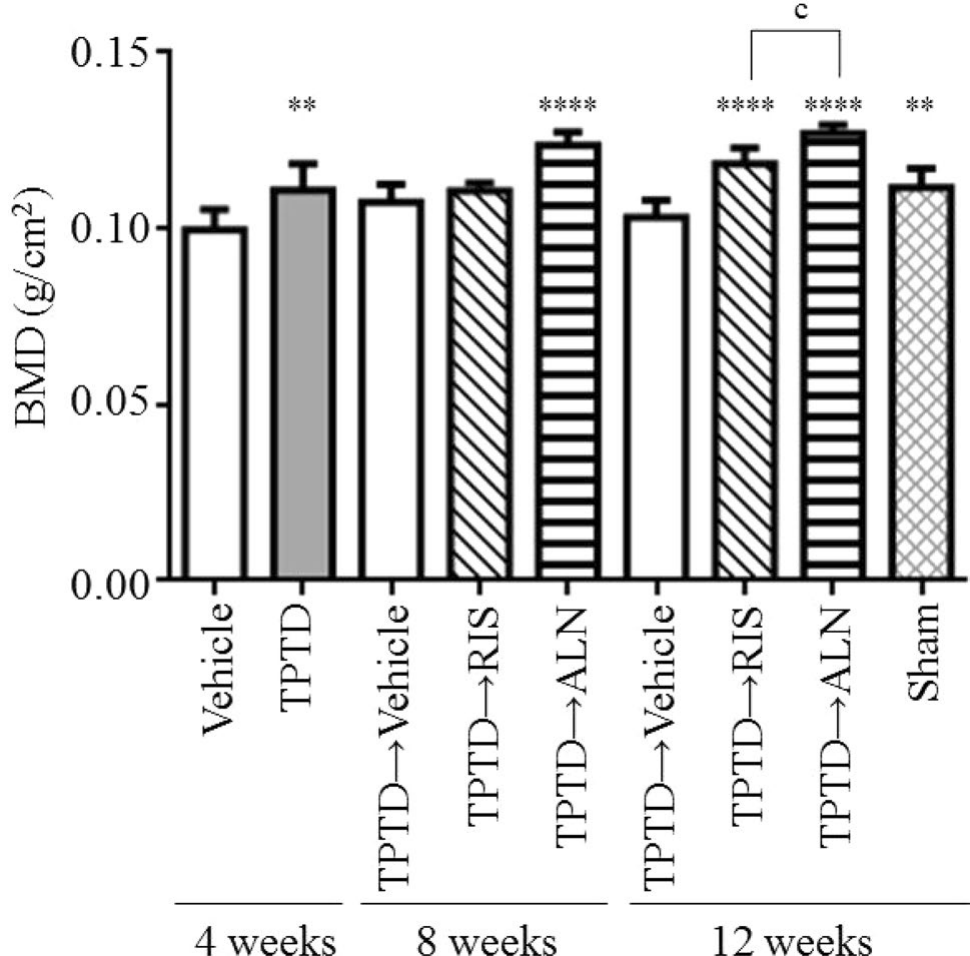
Effect of sequential treatment with bisphosphonates after teriparatide on lumbar vertebral BMD (L4). After ovariectomy, rats were treated with vehicle or teriparatide for 4 weeks. After teriparatide withdrawal, risedronate or alendronate was given for another 4 or 8 weeks. Lumber vertebrae were harvested from the rats at 4, 8, or 12 weeks for ex vivo BMD measurements by pDEXA. Values of lumbar vertebral BMD (L4) are shown in mean ± SD. *n* = 8. **p < 0.01 and ****p < 0.0001 compared with the vehicle-treated group at same time point.* c* *p* < 0.001 significant difference between risedronate and alendronate

As shown in Fig. 3, ex vivo BMD scans by pDEXA gave similar results to those obtained by in vivo DEXA. During the first 4 weeks, TPTD significantly increased BMD compared with vehicle. After TPTD discontinuation, sequential treatment with either ALN or RIS resulted in a greater increase in L4 BMD compared with vehicle at 12 weeks. However, at 8 weeks, significant difference from the vehicle was only observed with ALN. And BMD at 12 weeks was significantly higher in TPTD-ALN rats than that in the TPTD-RIS group (Fig. 3). Thus, at least effect on lumbar BMD appeared greater with ALN than RIS when used in sequential therapy after TPTD.

We also analyzed right tibia BMD and obtained virtually the same results: both ALN and RIS caused significantly greater increases than vehicle at 12 weeks, but ALN showed a slightly greater effect (data not shown).

### Mechanical Testing

**Fig. 4:**
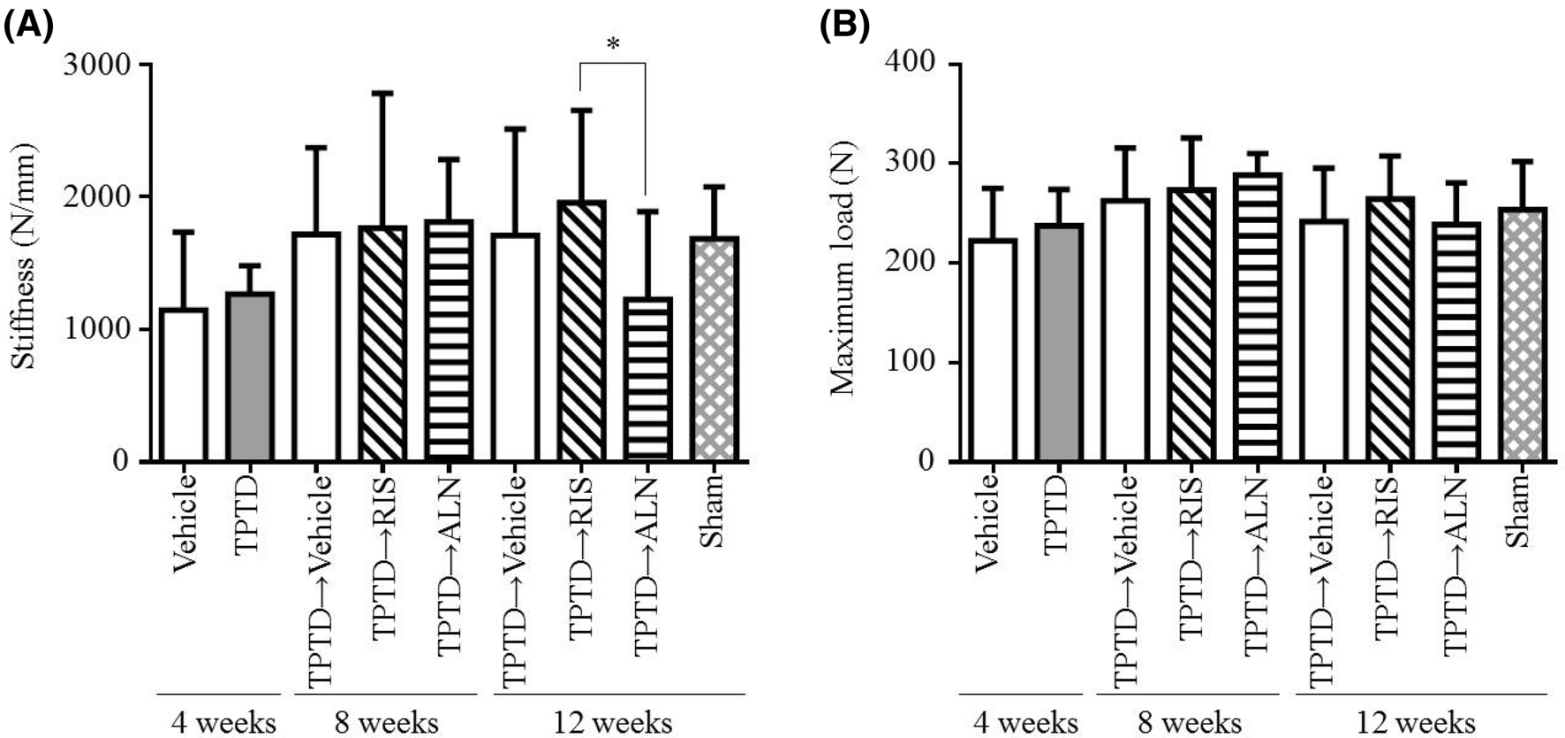
Effect of sequential treatment with bisphosphonates after teriparatide on **a** stiffness and **b** maximum load of lumber vertebra (L5). After ovariectomy, rats were treated with vehicle or teriparatide for 4 weeks. After teriparatide withdrawal, risedronate or alendronate was given for another 4 or 8 weeks. Lumber vertebrae (L5) were harvested from the rats at 4, 8, or 12 weeks and subjected to compression tests. Data are mean ± SD. *n* = 8. ^*^ *p* < 0.05 significant difference between risedronate and alendronate

Vertebral compression tests revealed that treatment with TPTD for 4 weeks caused no significant changes in stiffness (Fig. 4a). And sequential therapy with RIS did not significantly affect vertebral stiffness at 8 and 12 weeks, either. Interestingly, although ALN-treated rats showed higher BMD at 12 weeks, stiffness was significantly lower compared with RIS (Fig. 4a). As for maximum load, initial treatment with TPTD and a subsequent treatment with RIS and ALN resulted in no appreciable changes compared to vehicle at each time point (Fig. 4b). Three-point bending tests revealed no statistically significant effects of these drugs on femoral strength (Table 1).

**Table 1:** Effects of sequential treatment with bisphosphonates after teriparatide on femoral bone strength in ovariectomized rats

	4 weeks		8 weeks			12 weeks			
	Vehicle	Teriparatide	Vehicle	Risedronate	Alendronate	Vehicle	Risedronate	Alendronate	Sham
Stiffness (N/mm)	502.4 ± 43.0	490.2 ± 26.9	541.8 ± 47.9	575.3 ± 61.0	533.5 ± 53.2	575.0 ± 63.9	565.1 ± 76.8	567.8 ± 61.5	604.1 ± 32.9
Maximum load (N)	155.6 ± 16.6	157.0 ± 12.0	163.5 ± 10.0	172.9 ± 16.2	167.1 ± 10.4	172.0 ± 17.6	172.3 ± 12.3	173.7 ± 13.3	179.3 ± 13.1

After ovariectomy, rats were first treated with vehicle or teriparatide for 4 weeks, and then with vehicle, risedronate, or alendronate for another 4 or 8 weeks. After sacrifice, the right femur was obtained, cleaned of soft tissue, and tested for bone strength by three-point bending

Data are mean ± SD *n* = 8

### Histomorphometry of Lumbar Vertebrae

As shown in Table 2, histomorphometric analysis of lumbar vertebrae revealed that Ob.S/BS, OS/BS, BFR/BS, and MS/BS were significantly increased by TPTD compared with vehicle at 4 weeks, confirming enhanced bone formation. And at 8 and 12 weeks, discontinuation of TPTD caused a significant decrease in these parameters.

**Table 2:** Histomorphometric analysis of vertebra (L4) in ovariectomized rats sequentially treated with bisphosphonates after teriparatide

	4 weeks		8 weeks			12 weeks			
	Vehicle	Teriparatide	Vehicle	Risedronate	Alendronate	Vehicle	Risedronate	Alendronate	Sham
BV/TV (%)	24.42 ± 5.92	21.93 ± 4.35	21.19 ± 5.19	22.52 ± 6.80	24.15 ± 3.36	19.43 ± 4.14	25.24 ± 4.34	22.36 ± 5.24	23.32 ± 7.05
Tb.Th (μm)	68.18 ± 7.58	67.41 ± 8.42	72.54 ± 11.05	78.03 ± 16.12	73.46 ± 6.78	65.42 ± 5.07	77.24 ± 9.95	75.99 ± 14.10	65.53 ± 11.86
Tb.N (N/mm)	3.54 ± 0.54	3.24 ± 0.41	2.90 ± 0.37	3.21 ± 0.68	3.28 ± 0.30	2.95 ± 0.47	3.27 ± 0.42	2.95 ± 0.42	3.54 ± 0.82
Tb.Sp (μm)	220.42 ± 53.60	246.21 ± 47.81^*^	277.64 ± 51.99	244.20 ± 48.51^**^	233.46 ± 30.92^**^	281.60 ± 60.83	233.16 ± 40.93^**^	269.66 ± 51.16^$$^	232.06 ± 79.44
N.Oc/BS (N/mm)	0.18 ± 0.09	0.19 ± 0.14	0.20 ± 0.12	0.06 ± 0.04	0.08 ± 0.07	0.24 ± 0.16	0.07 ± 0.04^**^	0.10 ± 0.05^*^	0.35 ± 0.11
Oc.S/BS (%)	0.46 ± 0.22	0.53 ± 0.39	0.53 ± 0.34	0.15 ± 0.10^*^	0.23 ± 0.27	0.63 ± 0.48	0.22 ± 0.13^*^	0.27 ± 0.14	0.87 ± 0.25
Ob.S/BS (%)	2.58 ± 0.57	4.85 ± 2.12^*^	1.57 ± 2.00	0.26 ± 0.21	0.22 ± 0.11	1.35 ± 1.07^#, $$^	0.25 ± 0.21^**^	0.21 ± 0.15^**^	0.34 ± 0.21
OS/BS (%)	1.76 ± 0.54	5.06 ± 1.45^**^	2.01 ± 2.05	0.81 ± 0.55	0.54 ± 0.42	2.23 ± 2.68	0.14 ± 0.10^**^	0.20 ± 0.16^**^	0.48 ± 0.44
MS/BS (%)	28.24 ± 4.50	37.36 ± 5.85^**^	18.33 ± 6.08^$$$$^	12.07 ± 3.12^*^	12.16 ± 3.55^*^	21.32 ± 5.22 ^#, $$$$^	7.29 ± 3.37^**^	4.88 ± 1.13^**^	16.02 ± 4.38
BFR/BS (%/year)	12.14 ± 1.83	15.25 ± 3.23^*^	6.01 ± 2.24^$$$$^	3.18 ± 1.02^**^	2.71 ± 1.48^**^	6.12 ± 2.01^$$$$^	1.41 ± 1.78 ^**^	0.97 ± 0.89^**^	4.98 ± 0.88
MAR (μm/day)	1.19 ± 0.16	1.12 ± 0.14	0.92 ± 0.21	0.71 ± 0.11	059 ± 0.25^*^	0.78 ± 0.09^$$^	045 ± 0.49	053 ± 0.45	088 ± 0.13

After ovariectomy, rats were first treated with vehicle or teriparatide for 4 weeks, and then with vehicle, risedronate, or alendronate for another 4 or 8 weeks. After sacrifice, the 4th lumbar vertebra was removed free of soft tissue, processed, and analyzed for static and dynamic histomorphometric parameters

^*^ *p* < 0.05, ^**^ *p* < 0.01, ^***^ *p* < 0.001, and ^****^ *p* < 0.0001 compared with the vehicle-treated group at same time point. ^$$^ *p* < 0.01 and ^$$$$^ *p* < 0.0001 compared with risedronate at 8 or 12 weeks. ^#^ *p* < 0.05 compared with Sham group at 12 weeks. Data are mean ± SD. *n* = 8

Both RIS and ALN, administered after TPTD, significantly reduced parameters of bone turnover such as N.OC/BS, Ob.S/BS, and BFR/BS. As a result, these bisphosphonates improved bone volume and structural indices at 8 and 12 weeks, and a significant decrease was observed in Tb.Sp. Interestingly, RIS showed a significantly lower Tb.Sp compared with ALN at 12 weeks (Table 2). We also obtained similar results in the histomorphometric analysis of proximal tibia. Both bisphosphonates significantly suppressed turnover indices such as BFR/BS and increased BV/TV and structural parameters (data not shown). Tb.Sp at 12 weeks was lower in RIS compared with ALN: 569 ± 257, 209 ± 42, and 235 ± 58 μm in vehicle, RIS, and ALN, respectively. Thus, superior effect of RIS on trabecular quality appeared reproducible in two different types of bones.

## Discussion

Duration of TPTD treatment is limited to 2 years in clinical practice [12]. And TPTD effect seems at least in part reversible because discontinuation of TPTD results in significant bone loss. Thus, it is important and generally recommended for clinicians to sequentially treat patients with antiresorptives after TPTD therapy. There is, however, little evidence available for selection of specific post-TPTD therapy. Given the lack of such evidence, particularly, for bisphosphonates, we directly compared effects of two most commonly prescribed oral bisphosphonates, RIS and ALN, as a post-TPTD sequential therapy in OVX rats. The results demonstrated that both bisphosphonates were effective in terms of bone turnover suppression, BMD increase, and improvement of structural properties. As for the difference between the two bisphosphonates, ALN seemed stronger in turnover suppression and BMD increase, whereas RIS exhibited partially greater improvement in microstructure and strength.

In the current experiments, we adopted the same ratio of RIS and ALN doses as that clinically used (1:2). Although we do not have sufficient evidence or rationale for such a protocol in rodents, we were indeed able to observe some differences between RIS and ALN under such an experimental condition. First, BMD measurements by either in vivo DEXA or ex vivo pDEXA revealed greater effect of ALN than RIS in increasing BMD after TPTD therapy. This is consistent with the fact that in regular clinical doses, ALN showed a greater efficacy than RIS both in terms of turnover suppression and BMD gains in human subjects [38].

Contrary to a simple assumption that such BMD gains result in improved bone strength, the more robust increment in BMD by ALN did not translate into better mechanical properties compared with RIS. In fact, 8 weeks of treatment with RIS after 4-week TPTD treatment resulted in better stiffness in the compression test despite the lower BMD at the same time point, compared to TPTD-ALN sequential treatment. We are well aware that the results may not be definitively conclusive considering significant variations in such mechanical tests. Importantly, however, we were also able to demonstrate significantly lower trabecular separation at the histological level in RIS-treated rats. And the analyses of the bones in two distinct sites, vertebrae and proximal tibiae, showed consistent results. Thus, we believe that the results are suggestive of preferential effect of RIS on structural properties independent of BMD.

Although the mechanism is unknown, potentially superior effect of RIS on bone quality has already been recognized: clinical data have suggested that RIS appears more effective than ALN in preventing hip and non-vertebral fractures during the first year of therapy. Although no direct comparison has been made prospectively, RIS-treated subjects have a lower incidence of hip fractures than those treated with ALN for 12 months (0.37 vs. 0.58%) [30]. RIS is known to have less affinity to hydroxyapatite and more potency in inhibiting farnesyl pyrophosphate synthase than ALN. Such differences have been hypothesized to cause distinct distribution pattern and local effects. For example, a previous study demonstrated deeper penetration of RIS into the bone tissue compared to ALN [39]. And we have indeed demonstrated actual differences in the effects of RIS and ALN on micro architecture in calcium-deficient OVX rats [31]. Thus, in the context of post-TPTD therapy, it seems plausible that RIS exhibited a distinct effect from ALN on bone microstructure, most likely with complex interaction with residual effect of prior TPTD treatment.

There are limitations in this study. First, morphological analysis was only done with classical histomorphometry [40], as we were unable to analyze the bone with micro CT that has widely been used in recent years. Another limitation is that we used younger rats at 12 weeks of age compared to previous studies [34]. Our results should be interpreted with caution and may not be applied to older animals.

## Conclusions

In conclusion, we demonstrated that both RIS and ALN suppressed bone turnover, increased BMD, and improved structural properties in OVX rats when used for sequential treatment after TPTD. And we also found for the first time by direct comparison that ALN caused a stronger suppression of bone turnover and a more robust increase in BMD, whereas RIS treatment resulted in potentially better trabecular architecture and stiffness, suggesting preferential effect of RIS on bone quality. Whether such differences are indeed clinically relevant awaits fracture prevention studies by sequential treatment with different bisphosphonates after TPTD.
